# *Verbascum kurdistanicum* (Scrophulariaceae), a new species from Hakkâri, Turkey

**DOI:** 10.3897/phytokeys.52.5188

**Published:** 2015-07-08

**Authors:** Mehmet Fırat

**Affiliations:** 1 Yüzüncü Yıl University, Faculty of Education, Department of Biology, 65080 Van, Turkey

**Keywords:** New species, Hakkâri, Turkey, *Verbascum*

## Abstract

*Verbascum kurdistanicum* Fırat (Scrophulariaceae), is described and illustrated as a new species that is located in Hakkâri, Turkey. In this study, diagnostic morphological characters of this and closely related species (*Verbascum oreophilum* K.Koch and *Verbascum pyramidatum* M. Bieb) are discussed. Furthermore, distribution maps for the three taxa are provided.

## Introduction

*Verbascum* L. (Scrophulariaceae) includes about 360 species distributed throughout the world (Heywood 1993). This genus in Turkey includes about 245 species, 129 hybrids and 6 imperfectly known or doubtful records. Its endemism ratio is very high with 193 (79%) species restricted to Turkey (Huber-Morath 1978, Davis et al. 1988, Vural and Aydoğdu 1993, Karavelioğulları et al. 2004, 2006, 2008a, 2009a, 2009b, 2011, 2014, Karavelioğulları 2012, Sutorý 2001, 2004, Özhatay 2006, Kaynak et al. 2006, Yilmaz and Dane 2008, Bani et al. 2010).

*Verbascum* is divided into two sections (Murbeck 1933, Huber-Morath 1971): *Aulacospermae* Murb. and *Bothrospermae* (Murb.) Kamelin. The seed morphology of their members is the most important character which differentiates the sections. They are transversely corrugated and alveolate in sect. *Bothrospermae*, whereas the seeds are longitudinally corrugated in sect. *Aulacospermae*. Section *Bothrospermae* includes all Turkish *Verbascum* species.

The first revision of Turkish *Verbascum* for *Flora of Turkey* was carried out by Huber-Morath (1978). Thirteen species and six hybrids were later described (Vural and Aydoğdu 1993, Karavelioğulları et al. 2004, 2006, 2008a, 2009a, 2011, 2014, Karavelioğulları 2012, Sutorý 2001, 2004, Özhatay 2006, Kaynak et al. 2006, Dane and Yılmaz 2009, Dane and Yılmaz 2005, Yilmaz and Dane 2008, Karavelioğulları 2009b).

## Materials and methods

During field exploration in Hakkâri province, Turkey in 2011, an unusual population of *Verbascum* was discovered. At first glance, because of having corolla and capsule with branched eglandular hairs, glandular-hairy inflorescence and distinctly crenate lower leaves it seemed to be similar to *Verbascum oreophilum* and *Verbascum pyramidatum*. The specimens were cross-checked with the keys provided by Huber-Morath (1978, 1981) and the *Verbascum* accounts given in various relevant publications such as Fedchenko (1955), Feinbrun-Dothan (1978a, 1978b), Meikle (1985), Boulos (2009) and Ekim (2000). Herbarium specimens from VANF, GAZI, ANK, G and GB herbaria were also examined and compared. The threat category assessment of the new species was defined according to IUCN criteria (IUCN 2001).

## Taxonomy

### Verbascum kurdistanicum

Taxon classificationPlantaeLamialesScrophulariaceae

Fırat sp. nov.

urn:lsid:ipni.org:names:77148112-1

Figs 1 , 2

#### Type.

**TURKEY**. **C9 Hakkâri**: Berçelan Plateau, 37°40’57”N, 043°43’21”E, 2600–2800 m, limestone rocks and steppe, 21 July 2011, *M. Fırat.* 27584. (Holotype: VANF, Isotype: ANK, GAZI, HUB, VANF, E).

#### Diagnosis.

*Verbascum kurdistanicum* differs from *Verbascum oreophilum* and *Verbascum pyramidatum* by being biennial; having 8-30 (incl. petiole) × 2.5-4.5 cm, lanceolate, crenate basal leaves; linear-lanceolate calyx lobes; 4 stamens; two anterior filaments that are glabrous near apex; 10-15 × 6-8 mm, ovate to oblong capsule.

#### Description.

Biennial, 35–170 cm, densely stellate below, sparsely stellate, densely stalked glandular above. Stem robust, terete, erect, branched. Basal leaves 8–30 (inc. petiole) × 2.5–4.5 cm, mostly congested at base, densely rosulate, lanceolate, entire, distinctly undulate, obtuse, gradually attenuate at base. Cauline leaves 2.5–4 × 0.5–1 cm, oblong-lanceolate, entire, acute, decreasing in size towards the inflorescence racemose, ascending-erect. Bracts 2–3 × 1–2 mm, ovate-lanceolate, entire, acute, each bract with a solitary flower. Pedicels 5–10 mm. Bracteoles absent. Calyx 3–10 mm, divided almost to base, with linear-lanceolate acute lobes. Corolla 20–30 mm diam, yellow, tube 1–2 mm, with unequal and orbicular lobes, without pellucid-punctate glands, with sparsely stalked glandular, stellate outside. Stamens 4, 6–8 mm, filaments 5–6 mm, with purple-violet wool, two anterior glabrous near apex, anthers 1–2 mm, reniform. Ovary ovate. Style 5–7 mm, filiform. Stigma spathulate. Capsule 10–15 × 6–8 mm, ovate, densely stellate hairs, rarely soon glabrescent.

Flowering time June-July and Fruiting time July-August, *limestone rocks and steppe*, 2600–2800 m.

**Figure 1.**
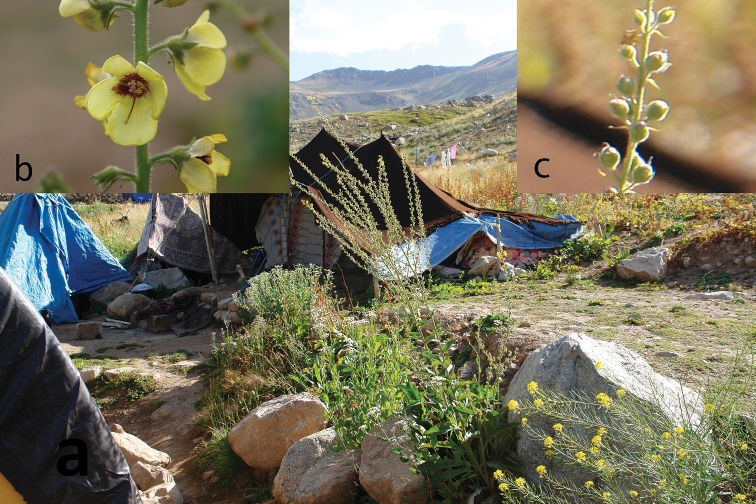
*Verbascum kurdistanicum* Fırat: **a** habit **b** corolla **c** capsule.

**Figure 2.**
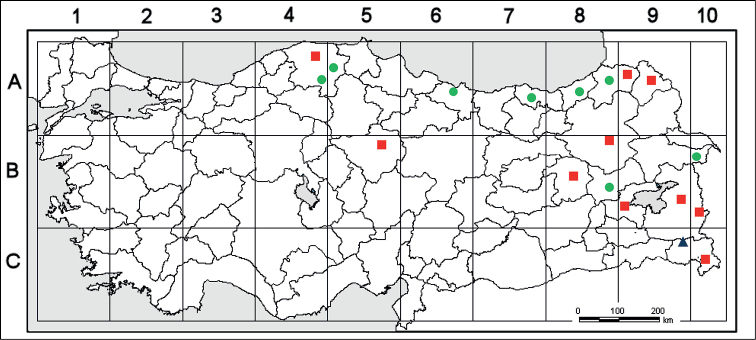
Distribution map of *Verbascum kurdistanicum* (▲) and also closely related species *Verbascum oreophilum* (■) and *Verbascum pyramidatum* (●).

#### Vernacular name.

In Hakkâri Province, indigenous people use the name “Masîjark” for *Verbascum* (Fırat 2013).

## Examined representative specimens:

***Verbascum oreophilum*: Turkey. C10 Hakkari**: Van-Hakkâri 113. km, c. 2400 m, 19 July 1956, alpine meadow, *H.Birand & K.Karamanoğlu 471* (ANK).

Other herbarium materials of *Verbascum oreophilum* and *Verbascum pyramidatum* which were collected from Turkey have been examined. VANF, GAZI, ANK, G and GB herbarium have been visited for representative specimens.

**Red list assessment**: The extent of occurrence for *Verbascum kurdistanicum* was less than 100 km^2^ (approximately between 10–20 km^2^). 304 mature individuals have been counted. This species was found in a single location. It grows in limestone rocks and steppe. Its habitat continues to decline because of agricultural activities and other local uses. Hence, the threat category of *Verbascum kurdistanicum* is suggested as CR [B1ab (i, ii, iii) + 2ab (i, ii, iii)].

## Results and discussion

Because of having 4 stamens, *Verbascum kurdistanicum* belongs to the group A according to the Flora of Turkey (Huber-Morath 1978, Davis et al. 1988). This group comprises 30 species with the addition of *Verbascum kurdistanicum*.

*Verbascum kurdistanicum* is morphologically similar to *Verbascum oreophilum* and *Verbascum pyramidatum* because of having the corolla and capsule with branched eglandular hairs; glandular-hairy inflorescence and distinctly crenate lower leaves, but differs from being biennial; having different basal leaves; stamen; flaments and capsule (Table 1).

**Table 1.** Diagnostic characters of *Verbascum kurdistanicum* compared with the related *Verbascum oreophilum* and *Verbascum pyramidatum*.

Characters	*Verbascum kurdistanicum*	*Verbascum oreophilum*	*Verbascum pyramidatum*
Plant	Biennial, 35–160 cm	Perennial, 60–160 cm	Perenial, 45–150 cm
Basal leaves	8–30 (incl. petiole) × 2.5–4.5 cm, lanceolate, crenate	10–40 × 5–20 cm, lanceolate-oblong to ovate-elliptic coarsely crenate or bi crenate, more rarely dentate-lobed	7–40 × 3–15 cm, lanceolate-oblong to obovate, coarsely bicrenate, crenata-dentate or weakly lobed
Calyx	lobes linear-lanceolate	lobes oblong	lobes oblong
Stamens	4	4-rarely 5	5-rarely 4
Filaments	two anterior glabrous near apex	two anterior woolly to anthers or glabrous near apex	all woolly
Capsule	10–15 × 6–8 mm, ovate to oblong	4–6 × 3–5.5 mm, ellipsoid-ovate or subglobose	4–8 × 3–5 mm, broadly elliptic to ovate

## Supplementary Material

XML Treatment for Verbascum kurdistanicum
